# Use of otoacoustic emissions to improve outcomes and reduce disparities in a community preschool hearing screening program

**DOI:** 10.1371/journal.pone.0208050

**Published:** 2018-12-10

**Authors:** Elizabeth Cedars, Hayley Kriss, Ann A. Lazar, Curtis Chan, Dylan K. Chan

**Affiliations:** 1 University of California San Francisco, Department of Otolaryngology–Head and Neck Surgery, San Francisco, CA, United States of America; 2 San Francisco Department of Public Health, San Francisco, CA, United States of America; 3 University of California San Francisco, Department of Epidemiology and Biostatistics, San Francisco, CA, United States of America; University of Leeds, UNITED KINGDOM

## Abstract

**Introduction:**

Hearing loss substantially impacts pediatric development, and early identification improves outcomes. While intervening before school-entry is critical to optimize learning, early-childhood hearing screening practices are highly variable. Conditioned play audiometry (CPA) is the gold standard for preschool hearing screening, but otoacoustic emission (OAE) testing provides objective data that may improve screening outcomes.

**Objectives:**

To compare outcomes of a community-based low-income preschool hearing program before and after implementation of OAE in a single-visit, two-tiered paradigm. We hypothesized that this intervention would reduce referral rates and improve follow-up while maintaining stable rates of diagnosed sensorineural hearing loss.

**Methods:**

We performed a cohort study of 3257 children screened from July 2014-June 2016. Department of Public Health data were analyzed pre- and post-implementation of second-line OAE testing for children referred on CPA screening with targeted follow-up by DPH staff.

Primary outcomes included referral rates, follow-up rates, and diagnosis of sensorineural hearing loss.

**Results:**

Demographics, pure-tone pass rates, and incidence of newly-diagnosed permanent hearing loss were similar across years. After intervention, overall pass rates increased from 92% to 95% (P = 0.0014), while only 0.7% remained unable to be tested (P<0.0001). 5% of children were unable to be tested by CPA screening but passed OAE testing, obviating further evaluation. Referral rate decreased from 8% to 5% (P = 0.0014), and follow-up improved from 36% to 91% (P<0.0001). Identification of pathology in children with follow-up increased from 19% to over 50%. Further, disparities in pass rates and ability to test seen in Year 1 were eliminated in Year 2.

**Conclusion and relevance:**

In a community setting, implementation of second-line OAE screening for CPA referrals reduced referral rates, increased identification of hearing loss, reduced outcome disparities, and improved follow-up rates. This study provides lessons in how to improve outcomes and reduce disparities in early-childhood hearing screening.

## Introduction

Hearing loss affects children’s cognitive, emotional, and social development, and educational, societal, and financial outcomes [1, 2]. Early identification and intervention through universal newborn hearing screening (NHS) has been shown to ameliorate these risks [3, 4]. However, as many as 36% of U.S. children referred on NHS are lost to follow-up [5]. The prevalence of permanent hearing loss is 1-3/1000 in newborns [6] and increases to 9-10/1000 in school-age children [2]; at least 50% of children with hearing loss at school-age are diagnosed after NHS [7]. Furthermore, large unbiased surveys suggest that the incidence of any detectable hearing loss in children is much higher, up to 20% [1, 2, 8, 9].

Early-childhood screening (EHS) can identify children with congenital hearing impairment lost to follow-up after a failed NHS [7], as well as those with late-onset, progressive, or fluctuating hearing loss, thus facilitating intervention prior to school entry. However, within the U.S. no routine postnatal EHS is recommended until the 4-year well-child check [10], or after public-school entry [2]. The approximately 1 million preschoolers enrolled in government-funded programs such as Head Start are required to have documentation of hearing screening [11]; however, no guidelines describe how this screening should be performed.

Conditioned play audiometry (CPA), in which children are conditioned to perform a specific action upon hearing a pure tone, is considered the gold standard method for pediatric hearing screening [2, 12]. However, the American Academy of Audiology (AAA) recommends that children with a chronological or developmental age under 3 be screened with otoacoustic emission (OAE) testing [2], an objective measure of cochlear function that does not require language comprehension or cooperation beyond allowing the placement of a probe into the ear canal. OAE screening is considered to be less accurate, sensitive, and specific compared to CPA [13].

The target age range for EHS, 2–5 years, encompasses the range where the ideal hearing screening method is unclear. While AAA guidelines recommend age 3 as the cutoff for CPA screening, children up to age 5 have been shown to be unable to cooperate [14]; many of these children may be at increased risk for hearing loss due to language, behavioral, or developmental differences and are thus poorly served by a behavior-dependent screen. Conversely, while objective OAE screening may increase the proportion of testable children by decreasing maturity-dependent participation, decreased sensitivity and specificity can overwhelm a screening program with false-positive referrals. AAA recommendations include implementation of a “2-tiered” screening system, involving rescreening after 6–8 weeks to reduce referrals for transient conditions [2]; however, such two-visit systems may be impractical in a community-based system owing to limited ability of screeners to return to sites for second visits. Previous studies have demonstrated variable outcomes with single and multi-step OAE protocols [13, 14, 15]; overall, reviews of preschool and school-age screening methods have generally concluded that CPA is the more sensitive and specific measure, while also noting variability in OAE studies that make direct comparison difficult [12, 13].

In this cohort study, we evaluated the outcomes of a community-based low-income preschool hearing-screening program conducted by the San Francisco Department of Public Health (SFDPH). By analyzing their transition from a CPA-based screening protocol to a novel hybrid CPA/OAE two-tiered, single-visit system, we aimed to compare outcomes and disparities of the two screening paradigms.

## Materials and methods

### Screening and follow-up protocols

Children attending preschool programs in the 2014–2015 and 2015–2016 school years underwent hearing screening performed by a single audiometrist in the Office of Childhood Hearing (OCH) at the SFDPH. Screening outcomes and demographic information were recorded by the audiometrist. Children who referred on at least one ear or who were unable to be tested for any reason were documented and their parents given notice of the result with the recommendation for additional evaluation by their primary care provider. In the 2014–2015 school year, screening was performed with CPA. The following year, initial screening was performed with CPA, with distortion product (DP) OAE screening as an immediate second screen for those who did not pass by CPA. Details on the screening program and methodology are provided in the Supporting Information.

Final audiologic and medical outcomes were documented by the OCH upon: 1) receipt of a completed medical documentation form given to parents upon notification of the screening outcomes; 2) direct verbal contact by an OCH and/or preschool staff member with the family or medical provider. All outcome documentation was stripped of personal health information and transmitted securely to UCSF at the conclusion of each school year for analysis. A complete description of the screening methods and follow-up protocols is included in the supplemental material.

### Definitions and statistics

Outcomes of screenings were documented as “Pass,” “Refer,” or “Unable to test” (UTT) at the time of screening. In Year 2, all Refer and UTT children were given OAE testing, leading to greater complexity of outcomes: all Refer children by CPA continued to be documented as Refer; all UTT children who were also UTT by OAE testing continued to be documented as UTT; those children who were UTT by CPA but Refer by OAE were counted as Refer; and those children who were UTT by CPA but passed by OAE were counted as Pass.

For the purposes of analyses, Refer and UTT children are categorized collectively as “Not Pass” (NP). Additionally, Pass and Refer children are collectively categorized as “able to test” (ATT). This allows separate analysis of two relevant outcomes of the hearing-screening protocol—the ability of the protocol to test the children (ATT vs UTT), as well as the referrals for further medical evaluation generated by the protocol (Pass vs NP).

Chi-square and Fisher’s exact tests, as well as logistic regression analysis both for the aggregate analysis and survey data, were performed by contract biostatisticians at the Clinical and Translational Science Institute at UCSF. Analyses were performed using SAS 9.4. This study was determined to be exempt from formal review by the Committee on Human Research at UCSF.

## Results

### Screening outcomes

**Fig 1:**
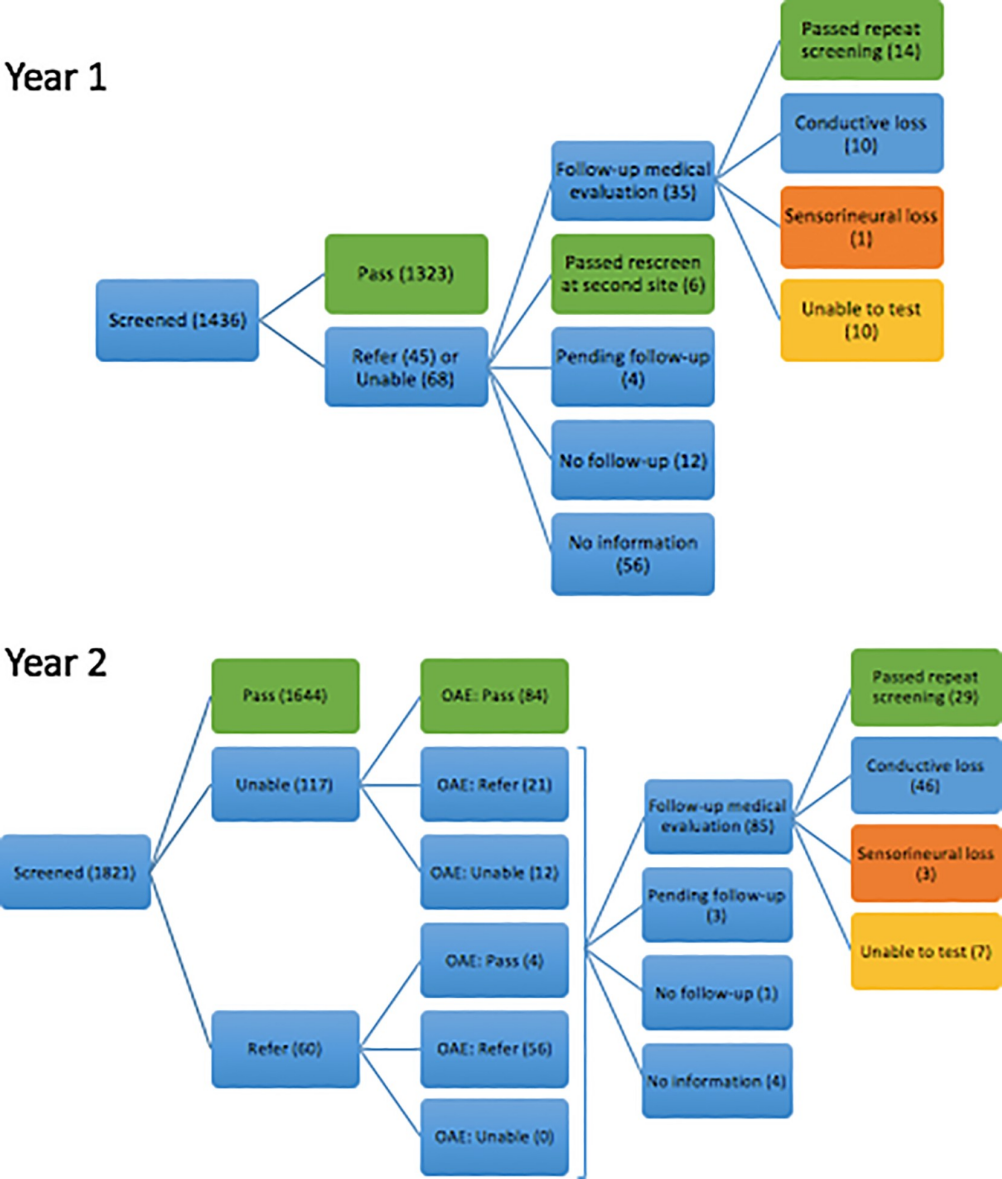
Flowchart of screening outcomes, year 1 (2014–2015) and year 2 (2015–2016). Children who ultimately passed their hearing screening are highlighted in green outcome boxes. Those diagnosed with sensorineural hearing loss are marked in orange boxes.

1436 children aged 2–6 were screened by CPA alone from July 2014-June 2015 (Year 1) and 1821 screened on a two-tiered single-visit CPA/OAE protocol from July 2015-June 2016 (Year 2). Screening outcomes (Table 1) demonstrate reduction in referrals (NP outcome) from 7.9% to 5.1% (P = 0.0014) in Year 2, largely attributable to children who were unable to be tested by CPA but passed upon OAE screening (4.6% of all children in Year 2). UTT rates were reduced from 4.7% in Year 1 to 0.7% in Year 2 (P<0.0001). Outcome flow map is shown in Fig 1.

**Table 1:** Screening outcome data by year. Year 1 (2014–2015) and Year 2 (2015–2016) hearing screening results. Data are presented as number of children with each outcome followed by percentage. P-values were calculated for change across years, for both Pass versus NP and ATT versus UTT.

	Year 1	Year 2	P-value
**Pass**	1323 (92.1)	1728 (94.9)	P = 0.0014
**Refer**	45 (3.1)	81 (4.5)	
**UTT**	68 (4.7)	12 (0.7)	P<0.0001
**Refer/Refer**	n/a	56 (3.2)	
**Refer/Pass**	n/a	4 (0.3)	
**Refer/UTT**	n/a	0 (0)	
**UTT/Pass**	n/a	84 (4.6)	
**UTT/Refer**	n/a	21 (0.9)	
**UTT/UTT**	n/a	12 (0.7)	
**ATT**	1368 (95.3)	1809 (99.3)	P<0.0001
**NP**	113 (7.9)	93 (5.1)	P = 0.0014

The demographic profile, including sex, age, primary language, ethnicity, and teacher concern about communication, was compared across years. Compared to Year 1, there were fewer Spanish- and Cantonese-speaking children, and fewer Asian children in Year 2 (Table 2).

**Table 2:** Demographic and independent variable data by hearing screening outcome 2014–2015 and 2015–2016. Demographic data are presented. Analyses comparing the number of children in each demographic category within years and across years was performed, with p-value noted to indicate statistical significance of differences in group demographics relative to reference (for within-year comparison) or between years.

		Year 1	Year 2	P-value	Type 3 P-value
**Overall No. (%)**		1436 (100)	1821 (100)		
**Sex**	Male	730 (50.8)	936 (51.4)	0.98	0.75
	Female	706 (49.2)	885 (48.6)	reference	
**Age**	2.1–3.0	63 (4.4)	108 (5.9)	reference	0.27
	3.1–4.0	464 (32.3)	592 (32.5)	0.086	
	4.1–5.0	661 (46)	816 (44.8)	0.051	
	5.1–6.0	248 (17.3)	305 (16.7)	0.067	
**Primary Language**	English	596 (41.5)	881 (48.4)	reference	<0.0001
	Spanish	397 (27.6)	406 (22.3)	<0.0001	
	Cantonese	357 (24.9)	368 (20.2)	<0.0001	
	Other	86 (6.0)	166 (9.1)	*	
**Ethnicity**	Asian	566 (39.4)	611 (33.6)	0.0011	0.0078
	Latino	346 (24.1)	456 (25.0)	0.17	
	Caucasian	183 (12.7)	284 (15.6)	reference	
	Other	341 (23.7)	470 (25.8)	*	
**Teacher Concern**	Speech	22 (1.5)	22 (1.2)	0.43	0.89
	Language	13 (0.9)	16 (0.9)	0.91	
	Hearing	6 (0.4)	8 (0.4)	0.96	
	None	1395 (97.1)	1775 (97.5)	reference	

*not reported, as includes a mix of multiple categories

### Follow-up

Follow-up among referred families improved after implementation of the two-tiered single-visit CPA/OAE protocol (Fig 1). While in Year 1, 56 families were unable to be contacted, in Year 2 only 4 families were not reached. In contrast to Year 1, when 12 contacted families did not seek follow-up, in Year 2 only 1 contacted family did not make an appointment with a primary care doctor as recommended, and 3 had pending appointments by the time of final contact. Completed follow-up rate among all 93 referred children at the conclusion of the academic year was therefore 91% (85/93), increased from 36% (41/113) in Year 1 (P<0.0001) (Table 3).

**Table 3:** Final outcomes of follow-up evaluations, year 1 and year 2. Outcomes of all referrals were documented. Those with follow-up were categorized by diagnosis, including passing rescreening without intervention (“passed rescreen”) and “unable to test”. Those without further evaluation were separated by reason: pending appointment (“pending”), no appointment sought (“no follow-up”), or no contact able to be established (“no information”).

	Year 1	Year 2
**Passed Rescreen**	20	29
**Conductive Loss**	10	46
**Ear Wax**	1	34
**Otitis Media**	7	10
**Required Tubes**	1	2
**Other Conductive**	1	0
**Sensorineural loss**	1	3
**Unable to test**	10	7
**No follow-up**	12	1
**Pending**	4	3
**No information**	56	4

### Diagnosis outcomes

We investigated the final diagnostic outcomes among all children referred through hearing screening (Table 3). Among the referred children with documented follow-up, the rate of identified sensorineural hearing loss (SNHL) increased from 1.8% (1/57) to 3.4% (3/89), but this difference was not statistically significant. The rate of identified conductive hearing loss increased from 17.5% (10/57) to 51.7% (46/89) (P<0.0001). Overall, pathology was found in 19.3% (11/57) of referred children with known outcomes in Year 1 and in 55.1% (49/89) in Year 2 (P<0.0001). Implementation of OAE screen therefore increased the rate of identified pathology among referred children with documented follow-up.

The overall prevalence of identified SNHL was 0.70 per 1000 screened children in Year 1, compared to 1.65 per 1000 in Year 2 (P = 0.6353). The prevalence of pathology increased from 7.66 per 1000 screened children in Year 1 to 26.91 per 1000 screened children in Year 2 (P<0.0001). Therefore, implementation of second-line OAE screening increased the identification of pathology among all screened children.

### Demographic analyses: Sex and age

**Fig 2:**
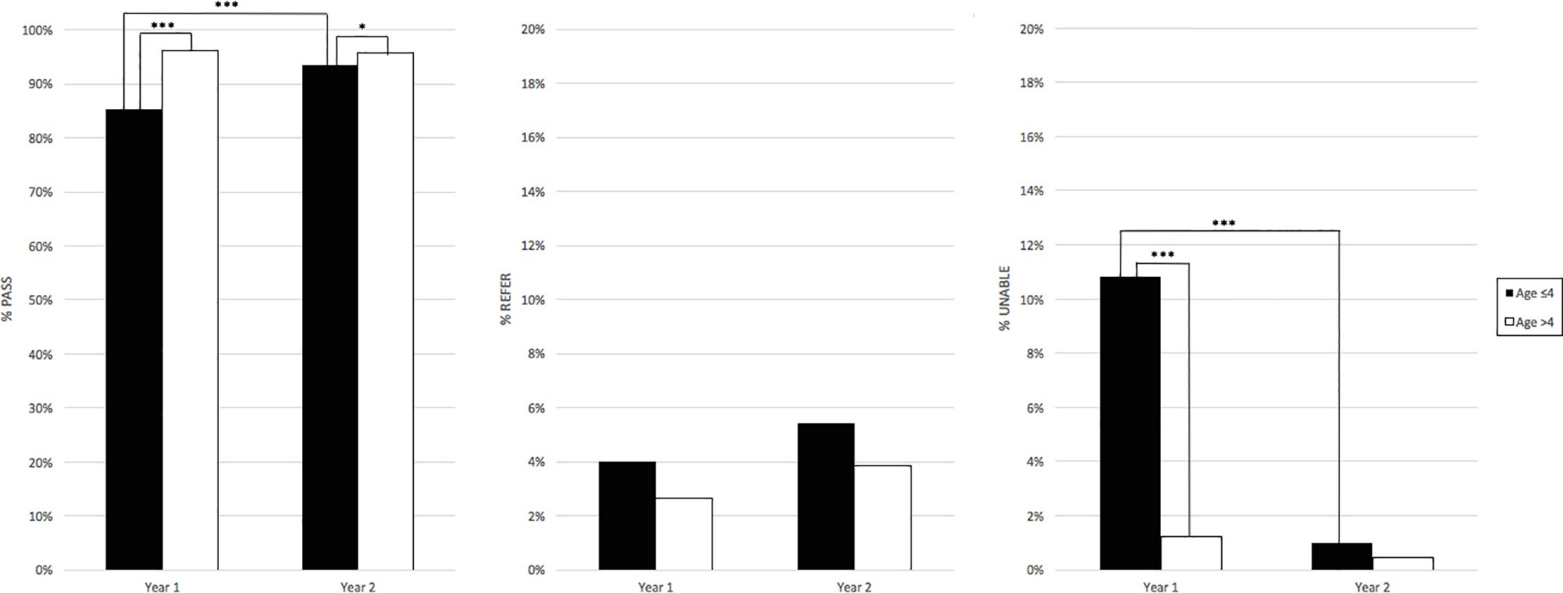
Outcome by age. Percentage of children ATT and passed (Pass), ATT but referred (Refer), or UTT (Unable) are indicated. In Year 1, children aged <4 were less likely to pass and more likely to be UTT than children >4. In Year 2, the difference in ability to test was eliminated. * p<0.05, ***p<0.0001.

Comparison of pass and ATT rates from year 1 to Year 2 are presented in Tables 4 and 5. Within each year, there were no differences in outcomes by sex. There was a statistically significant difference in outcomes by age, with children aged ≤4 significantly less likely to Pass as well as less likely to be ATT in Year 1 (Fig 2A). In Year 2, children aged >4 were still more likely to Pass than those age ≤4, but the two age groups were equally likely to be ATT (Fig 2). While children under 4 were more likely to be NP in both years, in Year 2 this was driven by Refer results rather than UTT, as there was no significant difference in the UTT rates between the two age groups in Year 2, whereas this difference was highly significant in Year 1 (Table 5). This reduction is accounted for primarily by the age 3.1–4 group, which had statistically significantly improved Pass and ATT rates compared to the >4 age group from Year 1 to Year 2 (Table 6).

**Table 4:** Pass/not pass outcomes comparison by demographic categories, by year and across years. Screening outcomes given as number of children (n). Statistical analyses (logistic regression) of outcomes by demographic categories with odds ratios and 95% confidence intervals, and analyses (Fisher’s Exact or Chi-Squared test) of each category across years are shown. Where type 3 p-values were not statistically significant, pair-wise analyses not presented.

		Pass VS Not Pass								
		Year 1				Year 2				Year 1 vs Year 2
		Pass	NP	OR [95% CI]	P value	Pass	NP	OR [95% CI]	P value	P value
Sex	**Male**	664	66	1.00		879	57	1.00		0.02
	**Female**	659	47	1.42 [0.96–2.09]	0.08	849	36	1.52 [0.99–2.30]	0.05	0.02
Age	**≤4**	449	78	0.25 [0.17–0.38]	<0.0001	655	45	0.65 [0.43–0.99]	0.043	<0.0001
	**>4**	874	35	1.00		1073	48	1.00		0.63
Language	**English**	531	65	1.00		843	38	1.00		<0.0001
	**Non-English**	792	48	2.0 [1.37–3.00]	0.0004	885	55	0.73 [0.48–1.11]	0.14	0.89
Ethnicity	**Caucasian**	171	12	1.00		274	10	1.00		0.18
	**Hispanic**	318	28	0.81 [0.41–1.63]	0.56	423	33	0.48 [0.24–0.98]	0.045	0.69
	**Asian**	514	52	0.71 [0.38–1.36]	0.30	572	39	0.55 [0.28–1.11]	0.10	0.07
	**Other**	320	21	1.09 [0.53–2.2]	0.82	459	11	1.53 [0.65–3.6]	0.33	
Concern	**Concern**	21	11	0.15 [0.07–0.31]	<0.0001	37	4	0.44 [0.16–1.22]	0.11	0.02
	**No Concern**	1293	102	1.00		1686	89	1.00		0.0007

**Table 5:** Ability-to-test comparison by demographic categories, by year and across years. Screening outcomes given as number of children (n). Statistical analyses (logistic regression) of outcomes by demographic categories with odds ratios and 95% confidence intervals, and analyses (Fisher’s Exact or Chi-Squared test) of each category across years are shown. Where type 3 p-values were not statistically significant, pair-wise analyses not presented.

		Ability to test VS UTT								
		Year 1				Year 2				Year 1 vs Year 2
		ATT	UTT	OR [95% CI]	P value	ATT	UTT	OR [95% CI]	P value	P value
Sex	**Male**	692	38	1.00		929	7	1.00		0.02
	**Female**	676	30	1.27 [0.78–2.07]	0.3339	880	5	1.29 [0.43–3.90]	0.65	0.02
Age	**≤4**	470	57	0.10 [0.06–0.20]	<0.0001	693	7	0.45[0.15–1.37]	0.16	<0.0001
	**>4**	898	11	1.00		1116	5	1.00		0.63
Language	**English**	565	31	1.00		876	5	1.00		<0.0001
	**Non-English**	803	37	1.19 [0.73–1.94]	0.48	933	7	0.78 [0.26–2.40]	0.66	0.89
Ethnicity	**Caucasian**	178	5	1.00		283	1	1.00		0.18
	**Hispanic**	330	16	0.62 [0.23–1.65]	0.34	453	3	0.69 [0.10–4.7]	0.70	0.69
	**Asian**	531	35	0.46 [0.18–1.15]	0.10	604	7	0.43 [0.07–2.5]	0.34	0.07
	**Other**	329	12	0.81 [0.29–2.3]	0.69	469	1	0.99 [0.10–9.6]	1.00	
Concern	**Concern**	25	7	0.16 [0.07–0.37]	<0.0001	40	1	0.18 [0.03–1.01]	0.051	0.02
	**No Concern**	1334	61	1.00		1764	11	1.00		0.0007

**Table 6:** Number of children per screening outcome by age.

		Pass	Refer	Unable	P-value (Pass/NP)	P-value (ATT/UTT)
**age 2.1–3.0**	**Year 1**	38	4	21		
	**Year 2**	99	6	3	<0.0001	<0.0001
**age 3.1–4.0**	**Year 1**	411	17	36		
	**Year 2**	556	32	4	0.0024	<0.0001
**age 4.1–5.0**	**Year 1**	635	17	9		
	**Year 2**	777	34	5	0.45	0.18
**age 5.1–6.0**	**Year 1**	239	7	2		
	**Year 2**	296	9	0	0.81	0.20

### Demographic analyses: Language and ethnicity

**Fig 3:**
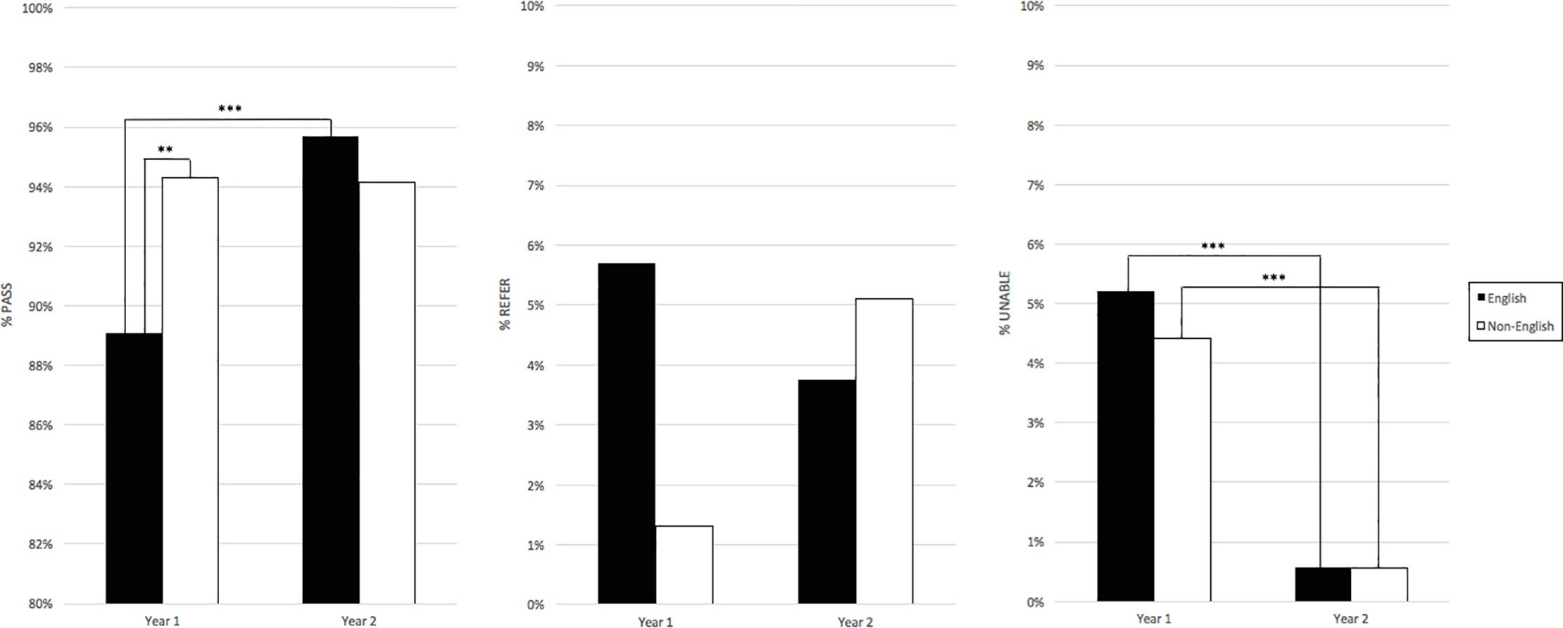
Outcome by primary language. Percentage of children grouped by primary language ATT and passed (Pass), ATT but referred (Refer), or UTT (Unable). In Year 1, primary non-English speakers were 2x more likely to pass than primary English-speakers, while all were equally likely to be ATT. This disparity resolved in Year 2. Improvements in UTT rates were seen in both English- and non-English-speaking groups from Year 1 to Year 2. ** p<0.001, ***p<0.0001.

In Year 1, children whose primary language was English were more likely to NP compared to Non-English-speaking children; they were all equally likely to be ATT (Fig 3). In Year 2, there was no difference in Pass or ATT rates among children with different primary languages.

Due to sample sizes, ethnic groups were clustered for analysis into Caucasian, Hispanic, Asian, and “Other”, which included African American, Native American, Pacific Islander, Multi-Racial, or Other. In Year 1 there was no relationship between ethnicity and hearing screening outcome. In Year 2, there were no differences in ATT rates among ethnicities, and no differences in Pass rates except among Hispanic children, who were less likely than Caucasian children to Pass. From Year 1 to Year 2 there was no significant change in Pass rates by ethnicity (Table 4).

### Demographic analyses: Concern for speech delay, language delay, or hearing loss

**Fig 4:**
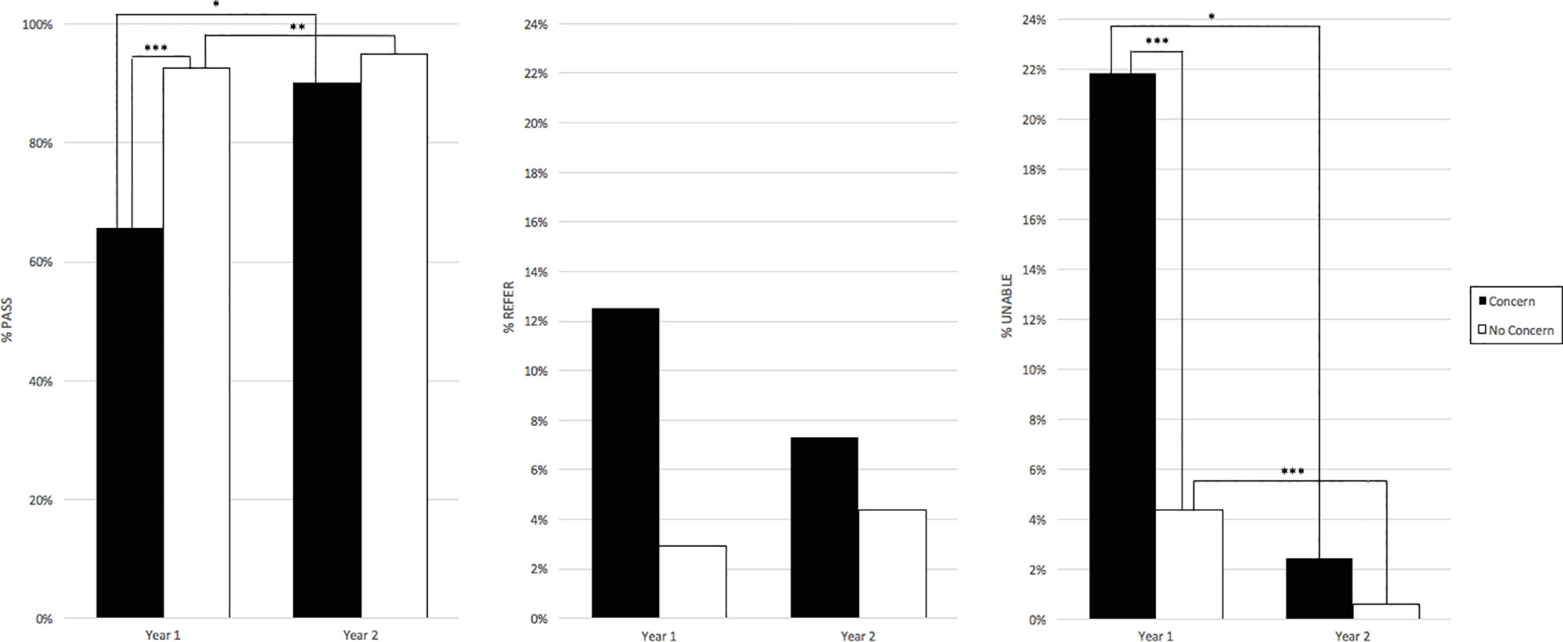
Outcome by teacher concern. Percentage of children grouped by teacher concern for speech, language, or hearing problems who were ATT and passed (Pass), ATT but referred (Refer), or UTT (Unable). Statistically significant differences between the Pass and UTT rates of children with concerns compared to those with no concerns were noted in Year 1 but not Year 2. Pass and UTT rates improved from Year 1 to Year 2 in children with concern and those with no concern.* p<0.05, ** p<0.001, ***p<0.0001.

In Year 1, 7 of the 11 NP children with concern for communication impairment were unable to be tested by CPA (Table 7). Children for whom there were any concerns were significantly less likely to Pass as well as less likely to be ATT. With the addition of OAE screening in Year 2, only 1 of 4 NP children with concern for speech, language, or hearing problems remained unable to be tested, and no statistically significant association was found with Pass or ATT rates (Fig 4).

**Table 7:** Number of children per screening outcome by concern for communication impairment.

		Pass	Refer	Unable
**Speech**	**Year 1**	13	4	5
	**Year 2**	18	3	1
**Language**	**Year 1**	5	2	6
	**Year 2**	13	2	1
**Hearing**	**Year 1**	3	2	1
	**Year 2**	6	1	1
**Total Concern**	**Year 1**	21	4	7
	**Year 2**	37	3	1
**No Concern**	**Year 1**	1293	41	61
	**Year 2**	1686	78	11

## Discussion

Preschool hearing loss presents a unique challenge. While it is critical to identify children with hearing loss during sensitive developmental periods, it is difficult to effectively screen and efficiently diagnose hearing loss due to variability in behavioral maturity among preschool children.

### Hearing screening outcomes

**Fig 5:**
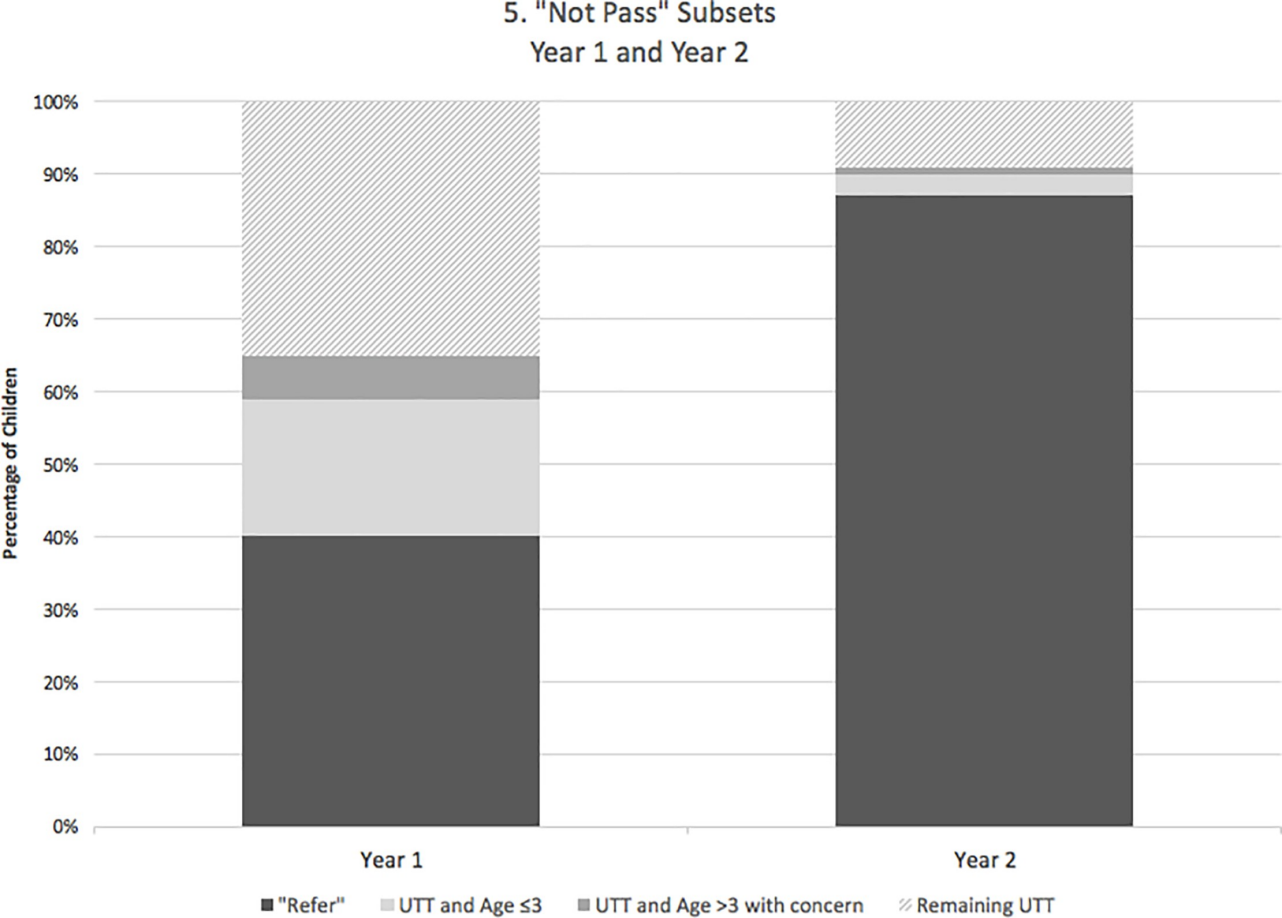
"Not Pass" subsets. All children referred in years 1 and 2 were categorized according to their hearing screening outcome, with those unable to be tested further divided by age and the presence of concern for delay.

Using a standard CPA-based hearing screening protocol in a community-based low-income network of preschools, we identified a high UTT rate (60.2% of referrals, comparable to prior studies [14]) with low confirmation of follow up (36.3%). Among all children who were referred for additional follow up in Year 1, 40% were able to be tested properly and did not pass the hearing screen, 25% were either under 3 years of age (19%) or had teacher concern for communication impairment (6%) and would therefore be recommended by AAA for alternative screening methods, and 35% were unable to be tested by appropriate CPA screening methods (Fig 5). To reduce the number of inadequately screened children, we introduced OAE testing as a backup for children who failed to pass initially with CPA screening. This method combines the gold-standard assessment of the entire auditory pathway with an immediate objective backup test.

With implementation of this two-tier, single-visit strategy, the overall referral (NP) rate dropped significantly, from 7.9% to 5.1%, due almost entirely to a large reduction in referrals for children who were unable to be tested by CPA alone. The referral rate in Year 2 was comparable to previous studies, including those utilizing more traditional 2-tier, 2-visit methods [16], indicating that over-referral for temporary pathology was unlikely to be a significant problem with our paradigm.

Importantly, the reduction in referral rates did not result in missed pathology. The incidence of confirmed SNHL was 1.65/1000 children in Year 2, a rate comparable to previously reported rates in preschool populations [12]. The rate at which SNHL was detected was unchanged from Year 1 to Year 2, and the prevalence of all pathology actually increased from 8 to 27/1000 children. Over 50% of referred children with documented outcomes had identifiable pathology, up from 19% in Year 1. This increase in pathology likely reflects improved detection rather than a rise in prevalence, as the two annual cohorts of children had comparable demographic profiles, and the 1-year interval between comparison groups was unlikely to allow a meaningful shift in population health status. The data therefore suggest that adding the OAE screen produced a more effective and efficient screening protocol, retaining detection levels of pathology while reducing overall referrals.

The rate of follow up increased substantially, from 36% in year 1 to 91% in year 2. While this improvement in follow-up rates cannot be definitively tied to the intervention due to the ecological nature of the study, it is possible that the reduced number of referrals in Year 2 could have improved the follow-up rate for children who did refer. DPH and preschool staff indicated that in Year 2 they had the same number of staff supporting a smaller number of referrals, which allowed them to focus more attention on tracking individual children’s outcomes. This could have led families to better understanding and support in seeking follow-up care, as well as improved documentation of follow-up. Further improvements in screening protocols, such as clarification of educational materials given to families and use of resources in additional languages, are currently in progress, and analysis after completion of this intervention would provide further insight into follow-up optimization.

### Disparities in hearing screening

In Year 1, there were significant disparities in hearing screening outcomes (Tables 4 and 5); in particular, younger children and those with concern for communication disorders were more likely to be unable to be screened by CPA alone. Though the AAA recommends OAE screening for children under 3, we found in our low-income community-based screening program that many 3-year-olds were unable to be tested; over half of children who were referred for inability to test were not covered by this AAA guideline (Fig 5). We found that, in fact, there was a significant difference in ability to test based on an age cutoff of 4 years. This difference was significantly attenuated with use of second-line OAE screening (Table 5).

According to 2007 guidelines from the Joint Commission on Infant Hearing [17], full audiologic testing is recommended in cases of teacher/caregiver concern for speech or language delay. We found that communication concerns are a significant predictor of poor screening outcomes (Fig 4). As with young children, those with communication concerns were more likely to be NP or UTT in Year 1. Though our cohort is limited in that there were overall few children with communication concerns who did not pass the hearing screen, ability to test was significantly improved with addition of OAE screening in Year 2.

Disparities in hearing outcomes relating to primary home language and ethnicity were more complex. In Year 1, we found no association between ethnicity and hearing screening outcomes, but did find, in contrast to prior literature, that non-English-speaking children were twice as likely to Pass hearing screens compared to English-speaking children, with no difference in UTT rates. One possible explanation for the language disparity without ethnicity difference may be the “healthy immigrant effect” [18], in which recent immigrants (non-Caucasian and non-English-speaking) tend to be healthier than impoverished non-immigrant populations (non-Caucasian and English-speaking). In Year 2, these language disparities were eliminated (Fig 3); however, ethnicity was newly identified as a predictive factor for referral–children of Hispanic ethnicity were more likely than Caucasian children to Refer. This association is consistent with findings from previous large cohort studies, which show that Hispanic Americans have a higher prevalence of hearing impairment compared to both Caucasian and African-American children [6]. Overall, the use of the second-tier OAE screen eliminated disparities in outcomes by primary language, and introduced a new disparity in outcomes among Hispanic children. Our data do not clearly suggest an underlying mechanism for this change, and more study is required to understand how language and ethnicity may impact community-based hearing screening strategies.

### Limitations

This study is limited in that it is an un-controlled, un-blinded, ecological prospective cohort study of screening practices. Thus, differences seen in Year 2 cannot be fully attributed to the intervention alone. However, consistency in the screened children demographics, childcare centers involved, and screening personnel between the control (pre-implementation of OAE backup screening) and intervention (post-implementation) groups minimize this limitation. Second, formal diagnostic audiograms were not performed in all screened children, making it impossible to calculate the sensitivity and specificity of the different screening paradigms; we cannot be certain whether either method resulted in false negative screens. Third, there are types of hearing loss that may be missed by reducing referrals with OAE testing: mild losses not identified by OAE screening; and auditory neuropathy spectrum disorder, in which cochlear function is intact but proximal signals remain impaired. These may be uncommon, but identification of these entities remains important. Finally, formal operational and cost analysis is beyond the scope of this current study, and findings may vary depending on local circumstances; future investigation may clarify these issues of importance to inform public health policy.

### Conclusions

The addition of an immediate second-line OAE screen to pure tone screening for preschool children improved both the effectiveness and efficiency of our community-based hearing screening program, and eliminated disparities in ability to test associated with age, language, and communication delay. The reduction in referral volume also corresponded to an improvement in follow-up rates, possibly by improving resource allocation. We therefore present this model as an effective way to provide single-visit, age-appropriate, efficient and effective hearing screening in community preschools.

## Supporting information

S1 File (DOCX)
