# Supplementary material for: Use of otoacoustic emissions to improve outcomes and reduce disparities in a community preschool hearing screening program
Source: PLoS One. 2018 Dec 10;13(12):e0208050. doi: 10.1371/journal.pone.0208050 (PMC6287830; doi:10.1371/journal.pone.0208050)
Supplement: S1 File — (DOCX) [file pone.0208050.s001.docx]

**Methodology**

**Pure tone screening**

In the 2014-2015 school year, children attending early childhood educational centers within the Child Healthcare Program were screened by an audiometrist in the Department of Public Health. This population consists primarily of children aged 3-5 from low-income families who were given reduced-cost schooling, with additional ages participating in preschool but not necessarily qualifying for financial assistance. Hearing screens were advertised on posters for several weeks prior to testing. Preschool staff were asked to let the screeners know of any children who were not to participate in screening – these included children whose parents did not want them to undergo testing and those who were known by the staff to have diagnosed hearing loss, who were excluded from screening. The number of excluded children was not recorded. The preschool staff were also asked whether they had concern for hearing loss, speech delay, or language problems for any of the children to be screened.

**Site Visit**

A single audiometrist visited each school site and screened all children present on the day of testing. Children were taken in groups of 2-3 to a quieter room or region of the classroom and conditioned to place a small toy in a cup upon hearing a tone. Translators (preschool staff speaking the child’s primary language) assisted in cases where the child did not understand the instructions in English. Children were screened individually at 1000 Hz, 2000 Hz, 3000 Hz and 4000 Hz. Tones were presented starting at 25 dB and increased at 5 dB increments until the child placed the block in the cup. If there was concern that blocks were not placed due to inattention, the tone was presented at 40dB to prompt the child, and then retested at 25dB. If no block was placed at all thresholds up to 60dB, that frequency was not tested further. Failure to place the block in the bucket at 25 dB presentation of all tones prompted referral for further medical diagnostic workup and audiologic testing. Children who were referred were recorded as “Unable” if they were not able to reliably comply with the instructions; they were marked as “Refer” for results of 30dB or greater in at least two frequencies in one ear, or 40dB or greater in any frequency in one ear.

**Notification**

On picking up their child, parents were given a letter which notified them of the screening results and a recommendation for further evaluation, and included a form for the documentation of the follow-up medical examination to be returned to the DPH. The letter was written in English, Chinese, and Spanish, and was given to the family by preschool staff speaking the child’s primary language.

**Follow-up**

In order to establish whether families sought follow-up evaluation after referrals, a combination of approaches was used. First, the notification form given to families of referred children included space for documentation by a health care provider of the follow-up assessment, with information about how to return the form to the DPH for documentation. Second, attempts were made by the audiologist by phone to reach the family of each referred child beginning 4-6 weeks after screening, both to determine follow-up outcomes and to administer a post-screening survey. The survey was developed by the authors with input from the audiologist and staff at the DPH. The survey is a 22-item, closed-set questionnaire requesting information about the family’s knowledge of the screening result, any follow-up healthcare appointments attended, reasons for not undergoing any step of follow-up evaluation, ease and clarity of the screening and follow-up process, and confirmation of primary language. It also offered further information about hearing loss and directions on how to initiate follow-up if none had been sought. Families were contacted either via a phone call from the audiometrist (using a translator for all non-English-speaking families) or in-person by a preschool employee using a paper survey document, depending on whether the childcare center allowed release of parental contact information. The families were contacted every 2 weeks after this first contact attempt. Three attempts at contact were made by the audiometrist (to the parent directly or to the school site). If no response was obtained after the three contact attempts, the family was determined to be lost to follow up and the case closed.

**Documentation**

Documentation was kept as follows:

-For each site, aggregate demographic data of the screened children was collected, including gender, age range (by 6-month intervals, compiled into 12-month intervals for consistency with year 2 data), primary language spoken, ethnicity, and concern for delay (hearing, speech, or language).

-For each site, screening outcomes were recorded as “Pass”, “Refer”, or “Unable”.

-For all referred children, individual-level data was recorded, including gender, age, primary language, ethnicity, and concern for delay, as well as screening outcome, parental survey responses (if any), notes on contact with parents/site and any results of medical assessments.

-As some children who saw their PCP after notification still required further evaluation for diagnosis, these children still in the midst of their workup were documented as “Pending” for the Final Outcome analysis, while being documented as “followed up” for the purposes of the Survey Outcomes documentation.

-Children whose parents completed the survey and had not had follow-up with their PCP were documented as “no follow-up” for the Survey Outcome. For parents who completed a paper survey, the audiometrist and school sites sought to communicate further with all of these parents. Some parents indicated that they would not arrange follow-up appointments; these were documented as “no follow-up” for the Final Outcome as well. Some were unable to be reached after the return of the survey; these were documented as “no information” for Final Outcome.

All data were collected by the audiometrist and deidentified, with subsequent secure transmission of data to UCSF for further analysis.

**Pure tone and second-line OAE screening**

In the 2015-2016 school year, a similar protocol was followed, with the following notable changes:

1. Children who received an “Unable” or “Refer” result on pure tone screening underwent distortion product otoacoustic emission screening. This was performed by first examining the external auditory canal with an otoscope to determine the presence of any occluding cerumen, and then fitting the child with foam earpieces and screening with a Bio-logic AuDx device. All settings on the device entailed DPOAE testing with default (“AJ”) settings according to the manufacturer’s instructions, described briefly as follows: Screening frequencies were 5000 Hz, 4000 Hz, 3000 Hz, and 2000 Hz, with F1 sound intensity 65 dB and F2 sound intensity 55 dB, F2/F1 ratio of 1.22, and Artifact Level 30. Pass/Refer criteria were frequency specific, with values of -6 at 5000 Hz, -5 at 4000 Hz, -8 at 3000 Hz, and -7 at 2000 Hz. Distortion product - noise floor difference was set to 6.

All children who had received “Refer” results on PT were given referrals for further evaluation, regardless of OAE results. If they had received an “Unable” result on PT testing but passed OAE screening, they were *not* referred for further assessment. If they received an “unable” or “refer” result with OAE testing, they were given the referral letter as in prior years. In summary, referrals were given for the following scenarios:

1. Pure tone results 30dB or greater in two frequencies of the same ear or 40dB or greater in any frequency of one ear, regardless of OAE result;
2. Children who were unable to complete the pure tone screening and did not pass the OAE (referred or unable to complete).
3. Documentation differed in that aggregate data for all screened children categorized age by 12-month intervals; results were recorded as “Pass”, “Unable PT/Pass OAE”, “Refer PT/Pass OAE”, “Refer PT/Refer OAE”, “Unable PT/Refer OAE”, “Unable PT/Unable OAE”; and otoscopy findings for children who underwent OAE screening were recorded.
4. Modifications to were made in the protocol for post-screening confirmation of follow-up. Families were contacted according to a schedule that provided earlier and more frequent contact attempts for screens with pure tone results of higher thresholds. The full schedule is outlined below. Contact was made by either the audiometrist or preschool, and documented verbally or by email. Additionally, the notification form given to parents whose child did not pass the hearing screen was also modified, creating separate forms for each language (English, Chinese, Spanish) to reduce the amount of text on the referral sheet.

| **Level** | **Screening Results at any frequency** | **Follow up call after screening** |
| --- | --- | --- |
| 1 - Mild | Appropriate response to a 26-40 decibel pure tone | 1. Call to site and/or parent 4-6 weeks  2. Call to site and/or parent 6-8 weeks  3. Call to site and/or parent 8-10  4. If no contact after 10 weeks, case is closed |
| 2- Moderate | Appropriate response to a 41-55 decibel pure tone | 1. Call to site and/or parent 4-6 weeks  2. Call to site and/or parent 6-8 weeks  3. Call to site and/or parent 8-10  4. If no contact after 10 weeks, case is closed |
| 3- Moderately Severe | Appropriate response to a 56-70 decibel pure tone | 1. Call to site and/or parent 2-4 weeks  2. Call to site and/or parent 4-6 weeks  3. Call to site and/or parent 6-8 weeks  4. Call to site and/or parent 8-10 weeks  5. If no contact after 10 weeks, case is closed |
| 4- Severe | Appropriate response to a 71-90 decibel pure tone | 1. Call to site and/or parent 2 weeks  2. Call to site and/or parent 4 weeks  3. Call to site and/or parent 6 weeks  4. Call to site and/or parent 8 weeks  5. Call to site and/or parent 10 weeks  6. If no contact after 10 weeks, case is closed |
| 5- Profound | Appropriate response to a 91+ decibel pure tone | 1. Call to site and/or parent 2 weeks  2. Call to site and/or parent 4 weeks  3. Call to site and/or parent 6 weeks  4. Call to site and/or parent 8 weeks  5. Call to site and/or parents 10 weeks  6. If no contact after 10 weeks, case is closed |

**If a child cannot complete pure tone and OAE screening they will be treated as a degree – 1

**Analyses**

All analyses were performed with the assistance of the biostatisticians at the Clinical and Translational Research Institute. Fisher’s Exact and Chi-squared tests were performed to compare demographic data with outcomes and as well as comparisons across years. Multivariate analyses were performed for all demographic data found to be statistically significant by chi-squared analysis.

Of note, when documenting concern for speech delay, language delay, or hearing loss, only the number of concerns was tracked, rather than the number of children for whom there were concerns. As individual-level data is available for all children referred for further evaluation (all refer and unable to test outcomes), the number of children for whom there were concerns can be counted directly. However, as there may be more than one concern per child and such data is not available for those children who passed, the total number of children for whom there was concern is not known.

In order to complete analysis, we assumed that for those children in the passing group, there was one concern per child. This overestimates the proportion of passing children with concerns for delay or hearing loss, and leads to the most conservative estimate of the effect of such concerns on pass rates. This assumption did not appear to meaningfully impact statistical analyses.
